# Utility of knowledge, attitude, and practice survey, and prevalence of dental caries among 11- to 13-year-old children in an urban community in India

**DOI:** 10.3402/gha.v6i0.20750

**Published:** 2013-04-30

**Authors:** Baranya Shrikrishna Suprabha, Arathi Rao, Ramya Shenoy, Sanskriti Khanal

**Affiliations:** 1 Department of Paedodontics and Preventive Dentistry, Manipal College of Dental Sciences, Manipal University, Mangalore, India; 2 Department of Public Health Dentistry, Manipal College of Dental Sciences, Manipal University, Mangalore, India

**Keywords:** dental knowledge, health practice, dental caries

## Abstract

**Background:**

The school oral health education program is believed to be a cost-effective method for promoting oral health. The KAP (knowledge–attitude–practice) model of oral health education is often the foundation of most health education programs.

**Objectives:**

To assess the existing knowledge, attitude, and oral health care practices among 11- to 13-year-old children and the association of knowledge with attitude, oral health care practices, and dental caries prevalence.

**Design:**

Cross-sectional design, involving 858 children studying in class seven at various schools in the city of Mangalore, India. The children were selected using stratified random sampling method. Prevalence of dental caries was determined using decayed, missing, and filled permanent teeth (DMFT) index. A self-administered questionnaire on self-care practices in oral health, knowledge, and attitude toward oral health care was filled by children. The association of different variables with knowledge was analyzed using binary logistic regression analysis.

**Results:**

The dental caries prevalence was 59.4%, and 54.5% had low knowledge. They lacked knowledge regarding use of fluoridated toothpaste and did not use them. Children with low knowledge had significantly higher odds of having DMFT≥1, not using fluoridated toothpaste, and being afraid of going to the dentist due to possible pain. There was no association of other oral health care practices and attitudes with knowledge.

**Conclusion:**

Oral health care practices and attitudes are not fully explained by knowledge, and other models of health education need to be considered.

During the past two decades, increasing levels of dental caries in children and adolescents have been observed in developing countries, in contrast to developed countries (1, 2). Among children, adolescents are particularly at higher risk for dental caries (3). Although adolescents have a basic knowledge of dental health, such as importance of proper brushing and diet in preventing dental caries, many fail to brush their teeth effectively and tend to consume cariogenic foods. They may underestimate health risks and tend to oppose their parents and teachers, making it the most difficult period for health education (4). At the same time, adolescence is a critical period as health practices during adolescence usually persist during adult years (3).

To overcome the high prevalence of dental caries in developing countries, the need for community-oriented preventive program is emphasized (5). Oral health education is an integral part of these programs. Oral health education is believed to be a cost-effective method for promoting oral health if done through schools, where all children irrespective of their socioeconomic status or ethnicity can be reached (6). The KAP (knowledge–attitude–practice) model of oral health education is often the foundation of most health education programs. According to this model, adequate oral health practices occur due to healthy attitudes which in turn develop due to proper knowledge (7). Tewari et al. observed that daily tooth brushing became more frequent after a community education program about oral hygiene (8). In other studies based on the KAP model of oral health education, the educational intervention significantly improved oral health practice (4, 9, 10). Therefore, as a first step, baseline studies need to be carried out on knowledge, attitude, and practices regarding oral health. However, the assumption that knowledge on oral health maintenance will modify children's attitude toward oral health and consequently change their oral health behavior is controversial (3, 11). Brukiene and Aleksejuniene in a systematic review of oral health promotion among adolescents found that intervention studies done using KAP model failed to achieve long-term success results in the absence of reinforcement programs (3). Hence, we carried out a study to assess the existing knowledge, attitude, and oral health care practices among adolescents in preventing dental caries in order to obtain a baseline data to carry out an organized school dental health program. The study also aimed to analyze the relationship between them so as to determine the relevance of the KAP model.

## Objectives

The objectives of the study were the following:

1. To determine the prevalence of dental caries in 11- to 13-year-old children attending schools in the city of Mangalore.
2. To determine the level of knowledge, attitude, and oral health care practices of these children.
3. To determine the association of knowledge with attitude, oral health care practices, and prevalence of dental caries within the study sample.

## Subjects and methods

The study was a cross-sectional epidemiologic survey of 11- to 13-year-old children (irrespective of whether they were in mixed and early permanent dentition period) studying in class seven at various schools in Mangalore, in the Dakshina Kannada district of Karnataka, India. Prior permission was obtained from school authorities and block education officers. Also, clearance from institutional ethical committee was obtained. Written consent forms were given to parents of children, and only those who gave consent were included in the sample. Children who had a history of chronic systemic diseases or mental disorders were excluded. A total of 865 children were invited to participate in the study. Finally, a total of 858 children studying in 14 different schools in Mangalore formed the study population. Schools were selected using stratified simple random sampling technique. Sample size for the study was calculated based on a previous study carried out in Mangalore among school children of the same age group (12). Schools of the city were divided into public and private schools and were randomly chosen to obtain a sample of 108 children from public schools and 750 children from private schools. This stratification was done to avoid bias as the earlier prevalence study (12) carried out in the same city had shown a very high prevalence of dental caries among children studying in public schools.

Dental caries prevalence was determined using the decayed, missing, and filled teeth (DMFT) index, all examinations being done by a single trained examiner (13). The examiner was calibrated prior to data collection and the inter-examiner agreement during calibration was measured using kappa statistic (κ = 0.86). The survey instrument was a pretested, self-administered bilingual questionnaire consisting of 18 questions. The questionnaire contained questions on self-care practices in oral health such as maintenance of oral hygiene, frequency of consumption of sugary foods, knowledge and attitude toward oral health care as well as sources of information on oral health. The validity of the questionnaire was ensured by mailing the prepared questionnaire to experts in the field. Changes were made in the questionnaire as per their suggestions. To test the reliability, a pilot study was carried out in a sub-sample of children, 10% of the original sample size, belonging to each of the randomly selected schools for the study. The ability of the children to understand the questionnaire was assessed and minor changes in terminologies were done based on responses. The questions were in Kannada (local dialect) and English. The reliability of translation to the local dialect was tested by back translation, done by a person with sound knowledge of the language, but who was not aware of the objectives of the study.

The questionnaire was given in the class room and filled by the children under the supervision of the class teacher to ensure that all questions were answered by the children. Interpersonal communications were not allowed, and children were informed of the importance of answering questions honestly.

Data were entered using SPSS package 11.5 and descriptive data were obtained. Chi-square test was used in statistical evaluation of bivariate frequency distributions. Multivariate binary logistic regression analysis was done to find the association of knowledge with attitude, oral health practices, and dental caries prevalence.

## Results

The results were obtained by dividing subjects into high and low knowledge groups. There were seven questions on knowledge regarding oral health care to prevent dental caries, with three answer options – true, false, and do not know (Table 1). Children who gave at least four correct answers were categorized as belonging to the high knowledge group and those with a score of three and below were considered to be of low knowledge. Overall, 44.5% had high knowledge and the remaining 55.5% had low knowledge. The study population consisted of 494 males and 364 females. The percentage of males with high knowledge (60%) was greater than females (40%) but the difference was not statistically significant (χ^2^ = 2.21, *p* = 0.27). The majority of children contended that the source of their knowledge on oral health were parents, (68.6%) followed by dentists (43.5%) and teachers (13.5%). The influence of television (10.7%), newspapers (8.9%), and friends (6.1%) was to a lesser extent in this regard.

**Table 1:** Percentage distribution of responses to questions on knowledge level

	True (%)	False (%)	Do not know (%)
Keeping natural teeth is important for general well-being	743 (86.6)	83 (9.7)	32 (3.7)
Natural teeth are better than false teeth	714 (83.2)	66 (7.7)	78 (9.1)
Brushing teeth can prevent decay	515 (60)	267 (31.1)	76 (8.9)
Eating and drinking sweet food does not cause decay	133 (15.5)	604 (70.4)	121 (14.1)
Regular dental check-ups are necessary	551 (64.2)	149 (17.4)	158 (18.4)
Flossing teeth can prevent tooth decay	255 (29.7)	82 (9.6)	521 (60.7)
Use of fluoride prevents tooth decay	171 (19.9)	126 (14.7)	561 (65.4)

Dental caries prevalence of the study population was found to be 59.4% implying that 40.6% was free of dental caries. The mean DMFT of the sample was found to be low (1.53±1.8), and the values ranged from 0 to 8. More than 70% of the children had DMFT between 1 and 3. The percentages of subjects with decayed, missing, and filled permanent teeth were 51.2%, 4.4%, and 14.8%, respectively. No statistically significant difference was seen between high and low knowledge groups with respect to the prevalence of dental caries (χ^2^=2.07, *p*=0.16) (Table 2).

**Table 2:** Distribution of knowledge among children according to dental caries occurrence

Dental caries	High knowledge (%)	Low knowledge (%)	N (%)
DMFT≥1	240 (61.7)	266 (56.8)	506 (59.4)
DMFT = 0	150 (38.3)	202 (43.2)	352 (40.6)
Mean DMFT	1.6±1.9	1.46±1.6	1.52±1.8

A large section of the study population brushed twice daily using a toothbrush and toothpaste, taking greater than 3 minutes for brushing. Sixty percent changed toothbrush every1–3 months. Bivariate analysis revealed no statistically significant differences with regard to oral hygiene practices between children with high and low knowledge. Most were not aware whether they used fluoridated/non-fluoridated toothpaste (78%) and the lack of awareness was significantly more in the low knowledge group (Table 3). The proportion of population which consumed cariogenic foods >4 times a day was around 10%. The proportion was marginally higher in children with good knowledge for most of the cariogenic foods but was not statistically significant (Table 4). More than 80% of children had visited a dentist in the past year. Visits to the dentist were significantly higher in children with high knowledge, and they mostly visited with a complaint of pain or for tooth cleaning. Children with low knowledge mostly visited the dentist for a check-up, due to cavities or food impaction. Overall, the proportion of the population that visited for a dental check-up was low (12%) (Table 5).

**Table 3:** Percentage distribution of various oral hygiene practices according to the level of knowledge

Variable	High knowledge (%)	Low knowledge (%)	N (%)	χ^2^	p
Tooth brushing frequency per day					
Never	0 (0)	2 (0.4)	2 (0.2)		
Once	63 (16.2)	90 (19.2)	153 (17.8)	5.26	0.32
Twice or more	327 (83.8)	376 (80.4)	703 (81.9)		
Time of brushing					
Morning only	70 (17.9)	98 (20.9)	168 (19.6)	2.30	0.26
Morning and night	308 (79)	358 (76.5)	666 (77.6)	2.68	0.40
Night only	2 (0.5)	1 (0.2)	3 (0.3)	3.80	0.27
After every meals	24 (6.1)	22 (4.7)	46 (5.3)	1.19	0.86
Material used for tooth brushing					
Tooth paste	376 (96.4)	446 (95.3)	822 (95.8)		
Tooth powder	10 (2.6)	12 (2.6)	22 (2.6)	1.93	0.85
Charcoal/others	4 (1.1)	10 (2.1)	14 (1.6)		
Type of toothpaste					
Fluoridated	89 (22.8)	59 (12.6)	148(17.2)		
Non-fluoridated	18 (4.6)	15 (3.2)	33(3.8)	18.85	<0.001*
Do not know	283 (72.3)	394 (84.2)	677(78.9)		
Use of tooth brush					
Yes	381 (97.7)	455 (97.2)	836 (97.4)	2.31	0.54
Finger/others	9 (2.3)	13 (2.8)	22 (2.5)		
Method of brushing					
Horizontal strokes	100 (25.6)	124 (26.5)	224 (26.1)	0.34	0.90
Up and down strokes	217 (55.6)	259 (55.3)	476 (55.5)	0.78	1.00
No systematic method	86 (22.1)	120 (25.6)	206 (24)	3.65	0.45
Time spent on tooth brushing					
< 3 minutes	141 (36.4)	159 (34.6)	300 (34.8)	5.86	0.27
> 3 minutes	247 (63.6)	301 (65.4)	548 (63.8)		
Frequency of change of tooth brush					
1–3 months	247 (63.3)	268 (57.3)	515 (60)		
4–6 months	65 (16)	76 (16.2)	141 (16.4)		
7–12 months	12 (3.1)	29 (6.2)	41 (4.8)	7.03	0.21
After one year	10 (2.6)	14 (3)	24 (2.8)		
Do not know	56 (14.4)	81(17.3)	137 (15.9)		

* p ≤ 0.05

**Table 4:** Frequency distribution of consumption of cariogenic foods according to knowledge level

Variable	Frequency	High knowledge (%)	Low knowledge (%)	N (%)	χ^2^	p
Biscuit	>4 times a day	43 (11)	34 (7.3)	77 (9)		
	1–4 times a day	165 (42.3)	196 (41.9)	361 (42.1)		
	2–4 times a week	101 (25.9)	149 (31.8)	250 (29.1)	9.1	0.14
	2–4 times a month	49 (12.6)	63 (13.5)	112 (13.1)		
	never	32 (8.2)	26 (5.5)	58 (6.6)		
Jam	>4 times a day	19 (4.9)	22 (4.7)	41 (4.8)		
	1–4 times a day	78 (20)	99 (21.2)	177 (20.6)		
	2–4 times a week	82 (21)	105 (22.4)	187 (21.8)	0.6	0.6
	2–4 times a month	76 (19.5)	105 (22.4)	181 (21.1)		
	never	135(34.6)	137 (29.2)	272 (31.7)		
Sweets	>4 times a day	49 (12.6)	43 (9.2)	93 (10.7)		
	1–4 times a day	111 (28.5)	114 (24.4)	225 (26.2)		
	2–4 times a week	100 (25.6)	128 (27.4)	228 (26.6)	7.43	0.19
	2–4 times a month	79 (20.3)	118 (25.2)	197 (23)		
	never	51 (13.1)	65 (13.8)	116 (13.5)		
Soft drinks	>4 times a day	31 (7.9)	28 (6)	59 (6.9)		
	1–4 times a day	77 (19.7)	104 (22.2)	181 (21.1)		
	2–4 times a week	80 (20.5)	92 (19.7)	172 (20)	2.4	0.8
	2–4 times a month	126 (32.3)	150 (32.1)	276 (32.2)		
	never	76 (19.5)	94 (20)	170 (19.8)		

* p ≤ 0.05

**Table 5:** Utilization of dental services by the children

Variable	High knowledge (%)	Low knowledge (%)	N (%)	χ^2^	p
Visited dentist in the past one year					
Yes	341 (82.8)	379 (84.7)	720 (83.9)	8.02	0.03*
No	49 (17.2)	89 (4.5)	138 (16.1)		
Reasons for visiting the dentist					
Pain	198 (50.8)	191 (40.8)	389 (45.3)	13.30	0.003*
Food gets stuck between teeth	16 (4.1)	24 (5.1)	40 (4.7)	12.38	0.004*
Cavities	64 (16.4)	87 (18.6)	151 (17.6)	13.76	0.003*
Tooth cleaning	88 (22.6)	81 (17.3)	169 (19.7)	13.23	0.002*
Check up	47 (12.1)	60 (12.8)	107 (12.5)	12.95	0.005*
Any other complaint	28 (7.2)	52 (11.1)	80 (9.3)	17.09	<0.001*

* p ≤ 0.05

The majority of children perceived that decayed teeth makes them look bad, and they would visit the dentist as early as possible when they see cavities in their teeth or have pain in the teeth. Children with low knowledge were significantly afraid of visiting the dentist and perceived the visits to the dentist as unpleasant (Table 6).

**Table 6:** Percentage distribution of attitude to knowledge level

Variable		High knowledge (%)	Low knowledge (%)	N (%)	χ^2^	p
Decay makes my teeth look bad	yes	330 (84.6)	406 (86.8)	736 (85.8)	1.20	0.57
	no	60 (15.4)	62 (13.2)	122 (14.2)		
Afraid of dentist due to possible pain	yes	113 (29)	171 (36.5)	284 (33.1)	8.57	0.02*
	no	277 (71)	297 (63.5)	574 (66.9)		
Experience at the dental visit	pleasant	291 (74.6)	309 (66)	600 (69.9)	14.53	0.005*
	unpleasant	99 (25.4)	159 (34)	258 (30.1)		
If you see cavities in your teeth, what do you do?	See the dentist for filling	291 (74.6)	358 (76.5)	649 (75.6)	1.95	0.58
	See the dentist only when in pain	76 (19.5)	79 (16.9)	155 (18.1)		
	Don't care	23 (5.9)	31 (6.6)	54 (6.2)		
	See the dentist immediately	299 (76.7)	349 (74.6)	648 (75.5)		
If there is pain in your teeth, what to do?	Take tablets	34 (8.7)	36 (7.7)	70 (8.2)	11.06	0.04*
	Brush teeth more often	33 (8.5)	54 (11.5)	87 (10.1)		
	Try to cope	24 (6.1)	29 (6.2)	53 (6.1)		

* p ≤ 0.05

Binary logistic regression analysis was carried out with knowledge as the dependent variable and prevalence of dental caries, oral hygiene practices, frequency of consumption of cariogenic food, utilization of dental services, and attitude as covariates to find the association of knowledge with other variables. Children with low knowledge had significantly higher odds of having dental caries (DMFT≥1), not using fluoridated toothpaste, and being afraid of going to the dentist due to possible pain (Table 7).

**Table 7:** Odds ratio for logistic regression between independent variables and knowledge scores

Variable	B	Odds ratio	95% CI of odds ratio	p
DMFT ≥1	0.320	1.37	1.02, 1.85	0.035*
Type of toothpaste	0.799	2.22	1.49, 3.31	<0.001*
Afraid of dentist due to possible pain	0.407	1.50	1.08, 2.07	0.014*

* p ≤ 0.05

## Discussion

In the present study, effort was made to understand the level of knowledge, attitude, and practices of oral health care for prevention of dental caries. The majority of the study population exhibited lack of awareness regarding use of fluoride, use of dental floss, and regular dental visits. A positive association between low knowledge and presence of dental caries was seen, which is self-explanatory. It is said that children with inadequate oral health knowledge are twice more likely to have caries (6).

Children with low knowledge were found to be afraid of visiting the dentist and perceive dental appointments as unpleasant and therefore tend to visit the dentist for curative rather than preventive purposes. Lack of awareness among children may be a direct outcome of parental awareness and attitude. Children who have not been exposed to regular dental visits tend to be more afraid of dental visits (14). There was no relation between knowledge and other variables except for presence of dental caries, use of fluoridated toothpaste and being afraid of dental visits, indicating that despite that awareness does not always lead to practice among adolescents. This indicates that a method of imparting knowledge with sufficient emphasis on benefits of oral health care practices may prove beneficial. There is an established tendency for selective perception and retention of information following education (15). Awareness depends also on initial beliefs, cultural and socioeconomic conditions, and so on (3, 6, 11, 16). Therefore, it appears that the association of knowledge, attitude, and practice of oral health cannot be understood simply based on the KAP chain and should consider other factors also (17).

The examinations of the dental caries status were done according to WHO standard criteria, making it possible to compare the data with similar studies. However, examination under daylight could have resulted in underestimation of dental caries (18). The information on oral health knowledge, attitudes, and behavior was collected by means of questionnaire, but this data collection method has its limitations. This method assumes that knowledge and behaviors are absolute, but under condition of uncertainty, the individual may be biased, leading to inflated positive responses (15). Further, this is a cross-sectional study and hence a definite cause and effect of low knowledge with the prevalence of dental caries or oral health practices cannot be established. However, the study gives a possible association of the existing knowledge in the study population with the study variables.

## Conclusions

The following were the conclusions of the study:

1. Low knowledge is associated with the presence of dental caries, non-use of fluoridated toothpaste, and fear of visiting dentist due to possible pain..
2. Higher knowledge may not always result in the right attitude and oral health care practices, and thus, the relevance of the KAP model in dental health education remains questionable.

Accordingly, other oral health education models based on psychology or social learning theories such as the Health Belief model and the Transtheoretical model may be considered. Future research should aim at finding the most effective method of oral health education which changes individual behavior and sustains it for a longer period.

**Key Messages**

Although there is a need to increase knowledge among adolescents through school dental education program, knowledge alone does not result in change in attitudes and practices. Hence, there is a need to shift from KAP model to other models based on psychology.
